# The inhibiting effect of the transcription factor p53 on dengue virus infection by activating the type I interferon

**DOI:** 10.18632/oncotarget.15352

**Published:** 2017-02-15

**Authors:** Yan-Ling Hu, Xiao-Shan Li, Shu Xiong, Qiang Ma, Dan Liu, Zhong-Quan Shi, Jing Tang, Xian-Cai Rao, Fu-Quan Hu, Guo-Li Li

**Affiliations:** ^1^ Department of Pathogen Biology and Immunology, Chongqing Three Gorges Medical College, Chongqing 404120, China; ^2^ Department of Microbiology, The Third Military Medical University, Chongqing 400038, China

**Keywords:** dengue virus, p53, apoptosis, interferon, cell apoptosis

## Abstract

To investigate the role of the transcription factor p53 in the course of the dengue virus (DV) infection. The human hepatocellular carcinoma cell strain HepG2 with a low expression level of p53 was built by using the retroviral-mediated RNA interference technology, and was detected by Western blot. The wild group and the interference group were respectively infected by the type 2 DV. The viral titration was detected by the Vero plaque assay, the viral multiplication was detected by the immunofluorescence, the cell apoptosis after virus infection was detected by FCM and the level of IFN-β was analyzed by ELISA. Compared to the wild group, the expression level of p53 in the interference group decreased significantly, which indicated that the HepG2 cell strain with the low expression level of p53 was successfully built. 24h after DV infection, the virus titration in the interference group was 100 times higher than that in the wild group. The result of the immunofluorescence showed that, the amount of green fluorescent cells in the interference group was significant higher than that in the wild group. It was indicated that the DV infection was inhibited by p53. However, 24h after DV infection, there was no significant difference in the amount of apoptotic cells in both groups. And the amount of IFN-β in the wild group increased 6 times. The DV infection was inhibited by the transcription factor p53 by activating type I interferon pathway other than promoting the cell apoptosis.

## INTRODUCTION

In normal cells, the transcription factor p53, with a low expression level, closely relates to the cell cycle, the DNA damage, the cell differentiation and the cell apoptosis [1]. Recent research shows that rats, with p53 gene knocked out, are more susceptible to the virus infection, which indicates that p53 plays an important role in the anti-virus infection [2]. Based on current studies, p53 plays an antiviral role in 2 ways. On one hand, at the early stage of virus infection, the viral multiplication can be inhibited by p53 through promoting the secretion of the interferon and the signal transduction. The human papilloma virus, the Kaposi's sarcoma herpesvirus and the poliomyelitis virus can infect the host by specifically disabling p53 [3]. On the other hand, at the late stage of virus infection, the viral multiplication can be inhibited by promoting the infected cell apoptosis. The dengue virus (DV) is the pathogen of the human dengue fever and the dengue hemorrhagic fever/the dengue shock syndrome (DHF/DSS). This virus can be transmitted by the intermediary aedes which threatens people health in the tropical and the subtropical regions [4]. However, the role of p53 in the DV infection still remains unknown. In this study, in order to further investigate the effect of p53 on the DV infection, the human hepatocellular carcinoma cell strain HepG2 with the wild type p53, and the HepG2 cell strain with the low expression level of p53 were respectively infected by DV.

## RESULTS

### The expression levels of p53 protein in HepG2 cell between two groups

**Figure 1:**
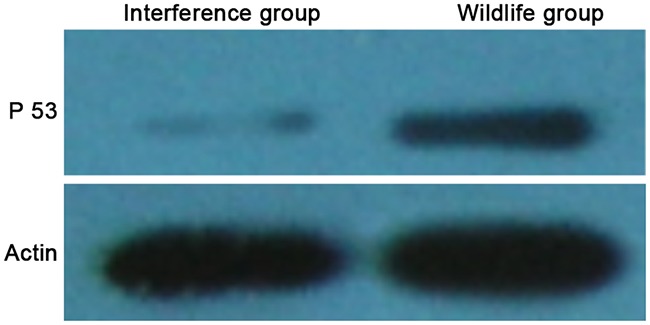
P53 protein levels detected by western blot

The WB result showed that the expression level of p53 protein in the interference group was significantly lower than that in the wild group (showed as Figure 1). It showed that the expression level of P53 protein in HepG2 cells was significantly decreased by the recombinant P53 siRNA, and the cell strain with a low expression level of P53 was successfully constructed.

### The apoptosis pathway of P53 in HepG2 cell

**Figure 2:**
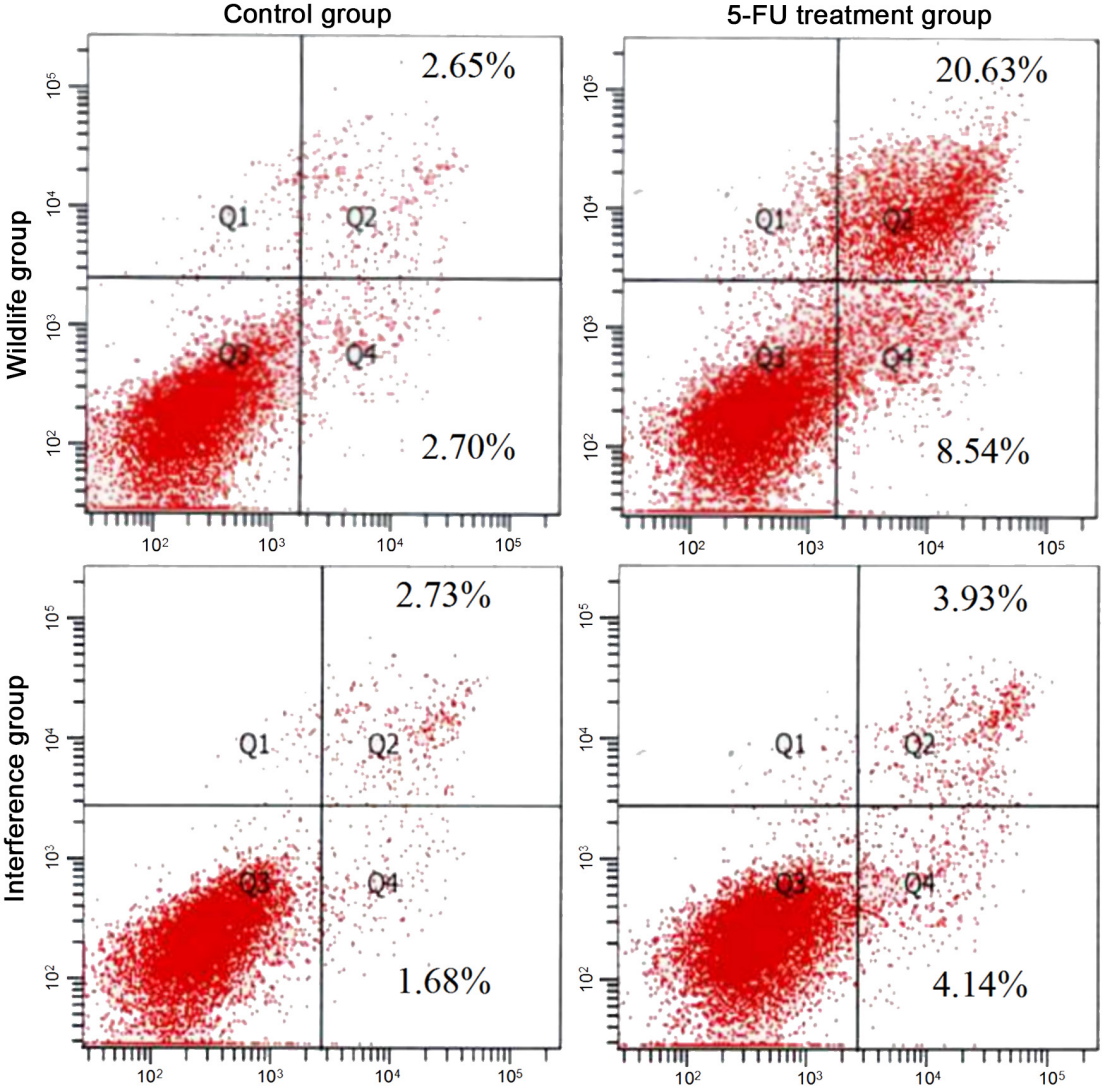
Apoptosis detected by FCM

The apoptosis was promoted by the 5-FU through activating the signal pathway of P53 [5]. The result of FCM showed that, before the processing of 5-FU, there was no significant difference between the HepG2 cells in the wild group and those in the interference group. 48h after adding 5-FU, the apoptosis rate in the interference group was 3.93%, which was remarkably lower than 20.63% in the wild group (showed in Figure 2). It showed that the cell apoptosis was promoted by 5-FU through the P53 pathway, and the high apoptosis rate in the wild group also indicated that the apoptosis pathway of P53 in HepG2 cells was normal.

### The viral titration

**Figure 3:**
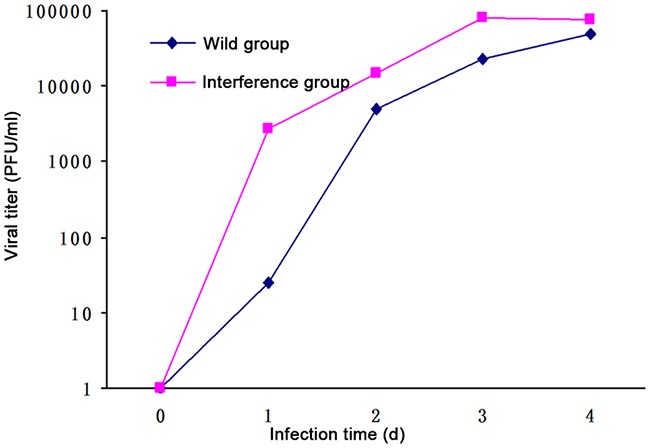
Viral titration detected by the plaque assay

The result of the plaque assay showed that: 1 day after DV infection, the viral titration in the interference group was 100 times higher than that in the wild group. However, there was no difference between the viral titration in the wild group and that in the interference group at 2 days, 3 days and 4 days after DV infection (showed in Figure 3). It indicated that the DV virus multiplication was inhibited by P53 which happened at the early stage.

**Figure 4:**
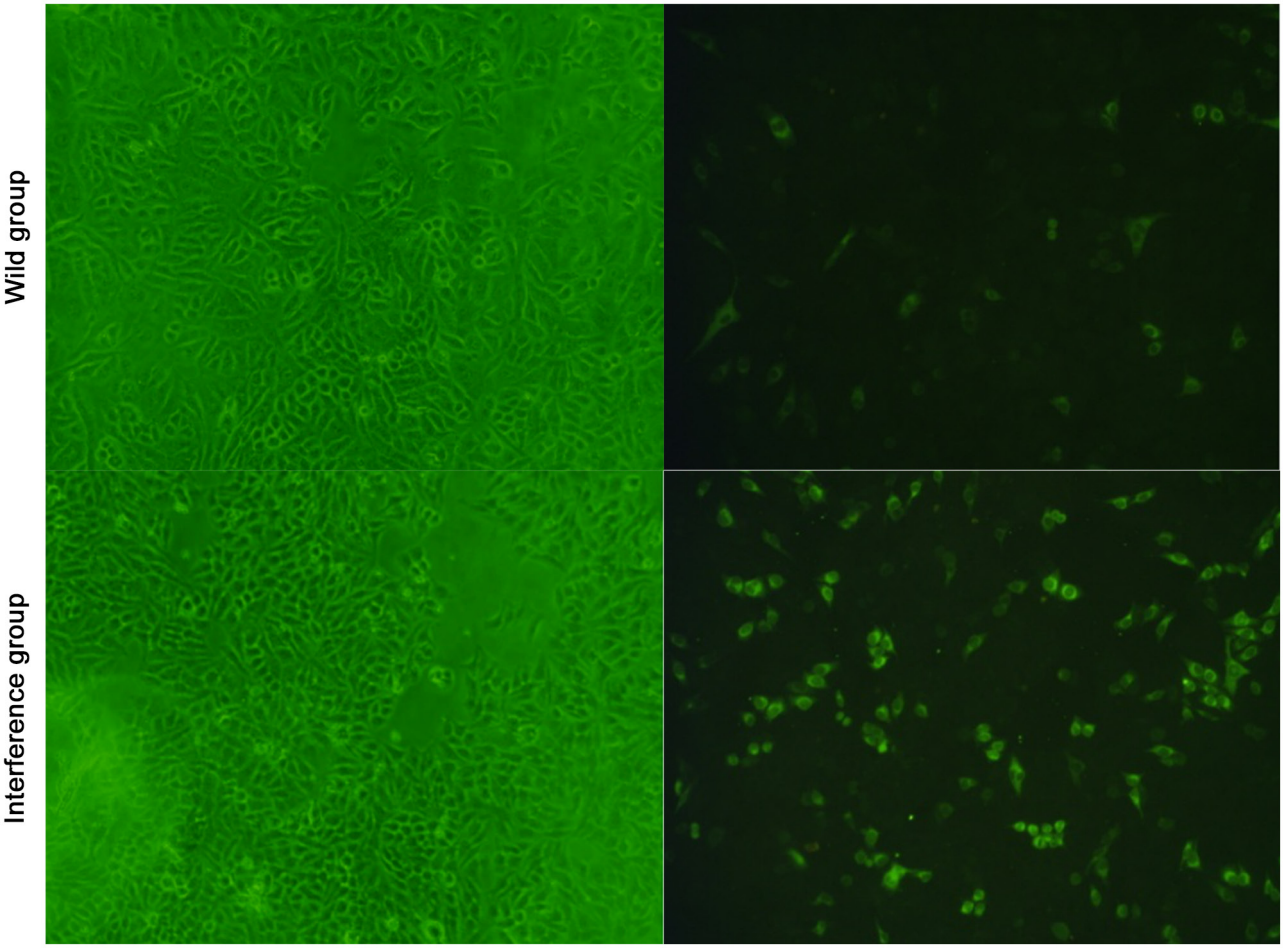
Viral multiplication detected by the immunofluorescence 100×

The result of the immunofluorescence showed that, when the amount of the cells was equally the same between the groups at 24h after DV infection, more virus antigens were found around the nucleus. And the amount of green fluorescent cells in the interference group was significant higher than that in the wild group (showed in Figure 4). It indicated that the virus multiplication was promoted by the low expression level of P53 in the interference group

### The cell apoptosis

**Figure 5:**
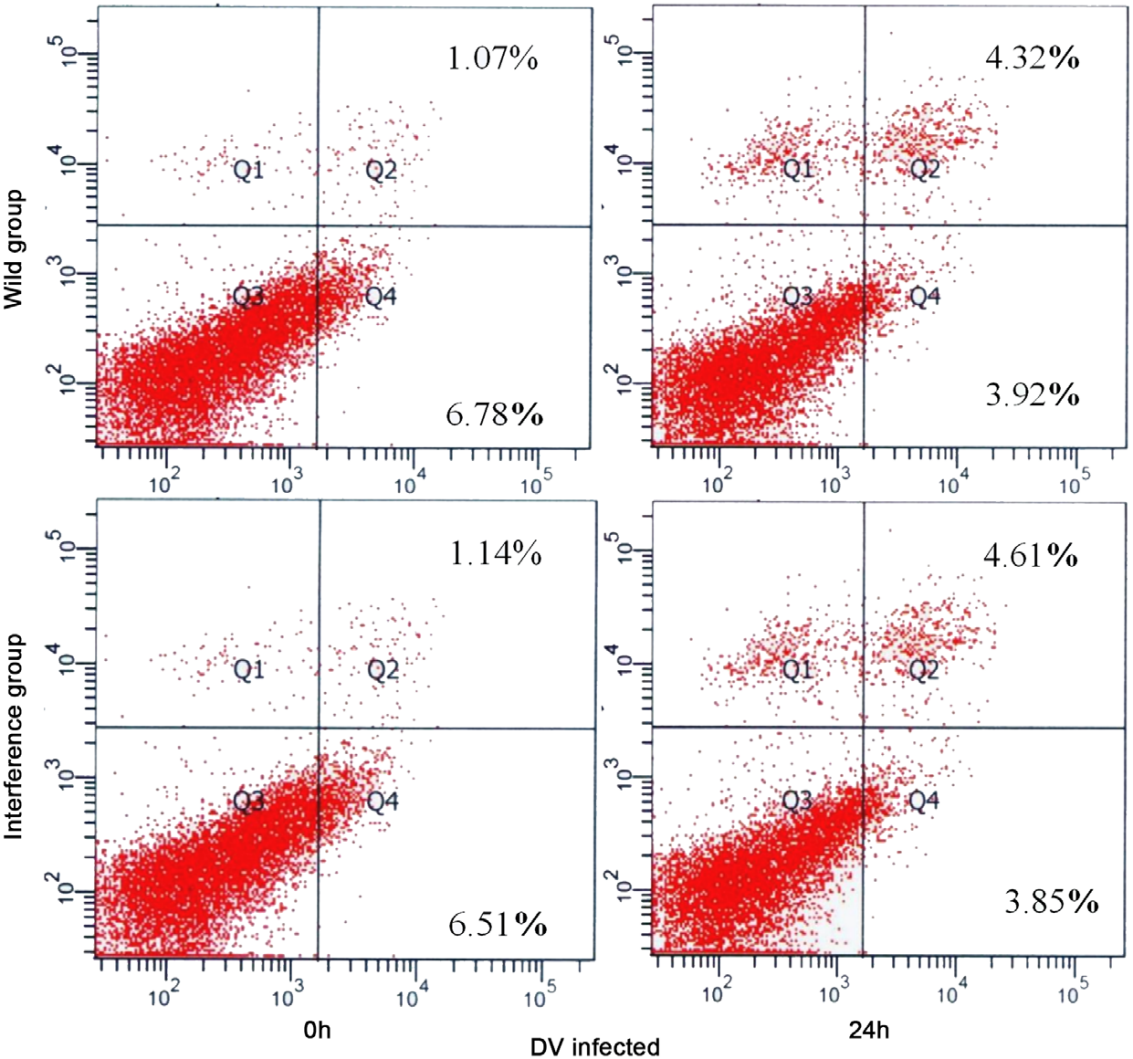
Apoptosis detected by FCM

At 24h after DV infection, there was no significant difference between the interference group and the control group (showed in Figure 5). The result showed that the antiviral effect of P53 was activated by other pathways instead of the cell apoptosis.

### The level of IFN-β

**Figure 6:**
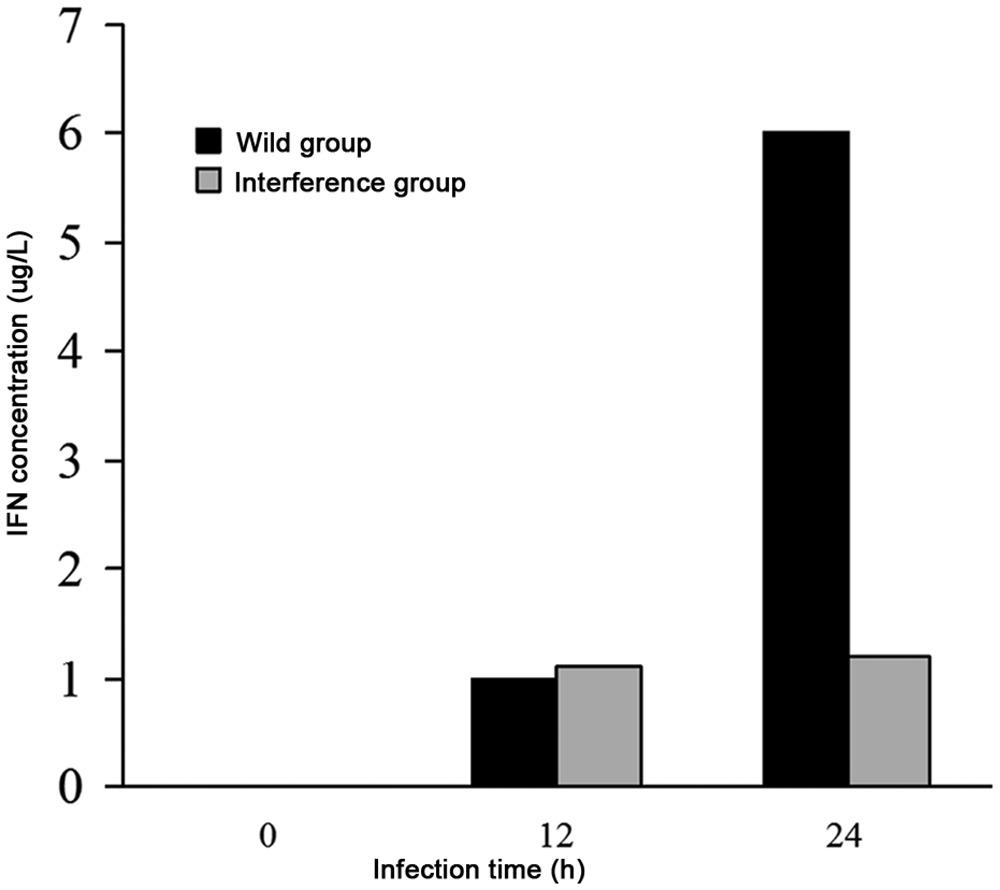
The levels of IFN-β analyzed by ELISA

The result showed that at 12h after DV infection, there was no difference in the levels of IFN-β in the wild group and the interference group. At 24h after DV infection, the level of IFN-β was 6 μg/L in the wild group, however, the level of IFN-β in the interference group kept unchanged (shown in Figure 6). It indicated that the secretion of the interferon in the virus infected cells was promoted by the high expression level of P53, and the interferon played an antiviral role at the early stage of virus infection.

## DISCUSSION

This study showed that, the viral multiplication was inhibited by p53 at the early stage of the DV infection, which was consistent with former literature. Researches show that, the amount of the viral particles released by the p53 deficient cell line is 9 times higher than that released by the wild p53 HepG2 cell line. It indicates that the release of DV particles is inhibited by p53 [6]. Furthermore, this study found that, in HepG2 the cell apoptosis was not promoted by the p53 pathway after DV infection. Reports, which study about the apoptosis mechanism induced by DV, reveal that the expression level of p53 in the DV infected hepatic cells doesn't increase, and p53 doesn't combine with DV protein. It shows that p53 doesn't participate in the regulation of the apoptosis in the DV infected hepatic cells, which is different from other virus infection [6]. In this study, 24h after DV infection, the amount of IFN-β in the wild group was significantly higher than that in the interference group, which indicated that the pathway of p53 antiviral effect related to the increased interferon secretion. Recent researches show that, the virus infection is inhibited by the independent stimulating effect of type I interferon which is mediated by the pattern recognition receptor RIG-I [7]. And IFIT3 has a protective effect on DV infection [8]. Specifically, the antiviral effect of the interferon pathway can be reversed by DV [9] through the interaction between the NS2B/3 protease and IκB kinase [10].

Typee I interferon (IFN-α and IFN-β) is secreted by the infected cells, which is induced by the virus infection. And the antiviral effect of non-infected cells can be triggered by the type I interferon. Therefore, the type I interferon is an important molecule of the antiviral innate immunity. The promoter of p53 can combine with the interferon-stimulated response elements (ISREs). Therefore p53 is considered as an activation-induced transcription factor in the downstream of type I interferon signal pathway [11]. In recent researches, the activated p53 can directly combine with the regulatory factor 9 (IRF9) of interferon pathway, which increases the expression level of interferon related genes and the antiviral effect [3]. The secretion of the interferon is promoted by the synergistic effect between the pattern recognition receptor RIG-I, the MDA-5 and the TLR-3, and the viral multiplication is inhibited accordingly [12]. Other reports find out that when DNA is damaged, the antiviral effect of type I interferon induced by p53 is strengthened by the stability of p53- dependent STAT-1 [13]. It indicates that STAT-1 is the targeted gene of the antiviral effect of p53. As for DV infection, the interaction mechanism of the p53 target gene and the interferon pathway still keeps unclear which needs to be further studied.

## MATERIALS AND METHODS

### Main materials and equipment

The HepG2 cell, the DV2 and the DV2 monoclonal antibody were provided by the microbiology laboratory of the Third Military Medical University. The HepG2 cell strain with a low expression level of P53, which was interfered by RNA, was also provided by this laboratory. The 5-FU was purchased from the company Sigma, the FBS and DMEM medium were purchased from the company Gibco. The mouse-anti P53 monoclonal antibody, HRP labeled goat anti-rat IgG and FITC labeled rabbit anti-rat IgG were purchased from the company Santa Cruz. The ECL kit was purchased from the company Thermo, and the human IFN-β ELISA detection kit was purchased from MLBIO Company. Annexin V-FITC/PI apoptosis detection kit was purchased from BD. The fluorescence microscope (Olympus Corporation), FCM (BD Company), Trans-Blot and ELIASA (BIO-RAD Company).

### The expression level of P53 detection

The total protein of the recombinant virus infected HepG2 cells and the normal HepG2 cells were respectively extracted, and the Bradford was used for the protein quantification (based on the Bradford protein quantification kit from Beyotime Biotechnology). Western blot was applied in this study. The total protein of the wild HepG2 cells and P53 siRNA HepG2 were isolated by SDS-PAGE and were transferred to the PVDF membrane by using the semidry method. 3h after sealing at room temperature, the anti-rat P53 primary antibody was added and both groups were incubated for overnight at 4°C. Both groups were washed by TBST 3 times and the dilute secondary antibody (HRP labeled goat anti-rat IgG) was added. The samples were vibrant incubated for 1h at room temperature, and were washed by TBST 3 times. And all samples were developed in the ECL kit.

### The apoptosis rate detection

The wild HepG2 cell suspension and siRNA HepG2 cell suspension with a low expression level of p53 were inoculated on the 6-well plate (5×10^5/^well). When the fusion was 80%, 200 μg/mL 5-FU was added into each well. 48h after incubation, the apoptosis rate was detected by FCM.

### Viral titration detection

p53 HepG2 both in the wild group and the interference group was incubated for 24h, and washed by the DMEM culture medium with 2% FBS. And DV2((MOI=1) was added and both groups were incubated for 60 min at 4°C. The virus bulk was discarded. After adding the DMEM culture medium with 2% FBS, both groups were incubated in 5% CO_2_ incubator at 37°C. At 1 day, 2 days, 3 days and 4 days after virus infection, the supernatant was respectively collected. The supernatant was diluted to 10^−1^~10^−6^ by the DMEM culture medium with 2% FBS. And 0.2ml of the supernatant was inoculated into the one-layer Vero cell. 1h after adsorption at 37°C, the virus bulk was washed off, and 1.5ml the DMEM culture medium with 1% methyl cellulose and 2% FBS were added into each well. When there was no development of the cell lesion, both groups were stained by the crystal violet. The PFU was recorded for calculating the viral titration.

### Viral multiplication detection

1×10^5^/ml cells were inoculated on the slides of 6-well plate, and after overnight cultivation the viruses (MOI=1) were inoculated into the slides. 12h, 24h and 48h after infection, the slides were fixed by the 4% paraformaldehyde PBS for 20 min. And PBS with 0.2% Triton X-100 was added for permeation for 5 min. The slides were sealed by 1%BSA PBS for 20 min, and later 50μl/pill (DV2 monoclonal antibody) was added. After overnight incubation at 4°C, the slides were washed by PBS 3 times, 5min/per time and the 50μl/pill second antibody (with FITC labeled rabbit anti-rat IgG) was added. After incubation for 1h at room temperature, the slides were washed by PBS 3 times, 5min/per time. After sealing by 40% glycerin, the slides were observed under the fluorescence microscope.

### The level of IFN-β analysis

100μl standard substance and 100μl sample were added into the corresponding reaction plate wells. The reaction plate wells were sealed after mixing for 30s, and were cultivated for 60min at 37°C. 100μl 1x Biotin was added into each well of the reaction plate which was washed 5 times. The reaction plate wells were sealed after mixing for 30s, and were cultivated for 60min at 37°C. 100μl 1x HRP was added into each well of the reaction plate which was washed 5 times. The reaction plate wells were sealed after mixing for 30s, and were cultivated for 30min at 37°C. 100μl TMB substrate was added into each well of the reaction plate which was washed 5 times. The reaction plate wells were sealed after mixing for 10s, and were cultivated for 15min at 37°C in the dark. 100μl stop buffer was added into each well of the reaction plate, which was sealed after mixing for 30s. At 450nm, the absorbance values were recorded within 30 min, which were used for calculating the level of IFN-β based on the standard curve.

### Statistical analysis

Statistical analysis was performed by using SPSS19.0 software. The data were analyzed by x ± s and the single factor analysis of variance was used. T test was applied to the comparison of multiple means and P<0.05 was considered as statistically significant.
